# Aseptic Ligatures Induce Marginal Peri-Implant Bone Loss—An 8-Week Trial in Rabbits

**DOI:** 10.3390/jcm8081248

**Published:** 2019-08-18

**Authors:** David Reinedahl, Silvia Galli, Tomas Albrektsson, Pentti Tengvall, Carina B. Johansson, Petra Hammarström Johansson, Ann Wennerberg

**Affiliations:** 1 Department of Prosthodontics, Institute of Odontology, Sahlgrenska Academy, University of Gothenburg, Gothenburg 405 30, Sweden; 2 Department of Prosthodontics, Faculty of Odontology, Malmö University, Malmö 205 06, Sweden; 3 Department of Biomaterials, Institute of Clinical Sciences, Sahlgrenska Academy, University of Gothenburg, Gothenburg 405 30, Sweden

**Keywords:** ligature induced peri-implantitis, dental implant, marginal bone loss, osseointegration, aseptic loosening

## Abstract

The clinical value of ligature-induced experimental peri-implantitis studies has been questioned due to the artificial nature of the model. Despite repeated claims that ligatures of silk, cotton and other materials may not induce bone resorption by themselves; a recent review showed that the tissue reaction toward them has not been investigated. Hence, the current study aimed to explore the hard and soft tissue reactions toward commonly used ligature materials. A total of 60 dental implants were inserted into the femur (*n* = 20) and tibia (*n* = 40) of 10 rabbits. The femoral implants were ligated with sterile 3-0 braided silk in one leg and sterile cotton retraction chord in the other leg. The tibial implants were ligated with silk or left as non-ligated controls. All wounds were closed in layers. After a healing time of 8 weeks, femoral (silk versus cotton) and proximal tibial (silk versus non-ligated control) implants were investigated histologically. Distal tibial (silk versus non-ligated control) implants were investigated with real time polymerase chain reaction (qPCR). The distance from the implant-top to first bone contact point was longer for silk ligated implants compared to non-ligated controls (*p* = 0.007), but did not vary between cotton and silk. The ligatures triggered an immunological reaction with cell infiltrates in close contact with the ligature materials, adjacent soft tissue encapsulation and bone resorption. qPCR further demonstrated an upregulated immune response toward the silk ligatures compared to non-ligated controls. Silk and cotton ligatures provoke foreign body reactions of soft tissue encapsulation type and bone resorption around implants in the absence of plaque.

## 1. Introduction

Dental implant therapy is a well-documented treatment for edentulism with an overall success rate of approximately 95% after 10 years [1]. A small degree of marginal bone resorption can often be observed during the first year of implant loading, probably due to tissue adaptation to the foreign material. Even though it is self-limiting in most cases, the marginal bone resorption sometimes progresses to the extent that the osseointegration becomes threatened. In a systematic review on implants with different surface types, Doornewaard et al., reported a 5% overall rate of implants with ≥3 mm of marginal bone loss after at least 5 years of function. They further indicated that smoking and a history of periodontitis yielded more bone loss [2]. With regards to treatment need, Albrektsson et al. reported that 2.7% of modern, moderately rough implants were either removed or subjected to other surgical procedures due to progressive marginal bone resorption during 7 to 16 years of follow up. [3].

In severe cases, continuous marginal bone loss may be locally detrimental to patients due to significant peri-implant bone defects that may impede future implant revision. Hence, animal models have been developed to study the onset and progression of marginal bone loss [4]. Such models have generally been based on the theory of peri-implantitis, which defines all marginal bone loss after osseointegration as solely a bacterial infection if coupled with bleeding from the peri-implant pocket in response to pocket probing [5].

The infectious peri-implantitis theory has been criticized for being narrow and exclusive of other potential causes for marginal bone resorption, such as poor implants, traumatic implantation or change of marginal conditions over time in response to wear products [6,7,8]. Furthermore, the marginal bone level has been shown to both increase and decrease around some implants over time, indicating a dynamic foreign body response to implants that may not require treatment, rather than a progressive infectious condition that demands intervention [9]. It has also been well-established that other types of endosseous implants that function in presumably sterile environments, such as hip and knee arthroplasties are equally susceptible to progressive bone loss and long-term failure (>10 years), as are transmucosal dental implants [10,11]. Aseptic loosening, caused by an immunological response to increasing amounts of wear debris at the bone-implant interface over time is believed to be the main cause of such late prosthetic joint failures [12,13].

In animal studies, undisturbed peri-implant plaque accumulation has resulted in negligible or absent marginal bone loss [14,15,16]. In order to speed up the process, a large majority of previous experimental peri-implantitis studies have therefore utilized sub-marginal ligatures of cotton, silk or other materials. Sub-marginal ligation has commonly resulted in significant bone loss after a few months, especially when the ligatures were replaced or added to in number every few weeks [17]. The ligature method aims to mimic infectious, clinical peri-implantitis and several authors have claimed that the utilized ligatures merely act as carriers of bacteria, without capacity to induce bone resorption by themselves [4,18,19]. In one of the very first ligature studies on implants, Lindhe et al. referred to a study that used silk ligatures in the periodontal tissues (natural teeth not implants) of germ free and conventional rats from 1966 to support this claim; however, none of the rats in either group lost any bone in that study [20]. The validity of the claim was further questioned in a recent systematic review by our group, in which we failed to identify any attempts to prove that ligatures cannot induce peri-implant bone resorption by themselves. Along with previous reviews on the method, we concluded that it remains unknown whether bone resorption can be induced by a foreign body reaction to the ligature materials themselves or the tissue trauma that results from their insertion [17,21,22]. An eventual capacity for ligatures to induce bone resorption in absence of bacteria would cast serious doubt on the clinical value of the method, especially when considering that ligation is an artificial manipulation that does not mimic any clinical condition, with the possible exception if someone unintentionally leaves a retraction chord in a peri-implant pocket, which, however, must be regarded a most unusual clinical error. Hence, a validation of the infectious model of explanation provided for the ligature method is warranted. 

The aim of the current study was to evaluate the capacity of aseptic ligatures to induce peri-implant bone resorption in rabbits. We chose silk ligatures due to their common use in other small animals, i.e., rodents, as well as existing, relevant real time polymerase chain reaction (qPCR) data from one of these studies [23] and an ongoing aseptic silk ligature trial on rats, by another group at our faculty. The hard and soft tissue reactions against the silk ligatures were evaluated for tibial implants, with histological methods and also selected qPCR markers in order investigate the immunological activity of adjacent cells, as well as the local bone reactions. Furthermore, since no comparisons of different ligature materials have been published previously according to our knowledge, a histological comparison of silk and cotton ligatures was also performed on femoral implants. Our null hypothesis was that marginal ligatures should not be able to induce marginal bone loss in the absence of a bacterial plaque, as repeatedly claimed in previous ligature induced peri-implantitis studies [4,18,19].

## 2. Experimental Section

### 2.1. Implants

The implants used (Ospol Regular, Malmö, Sweden) were turned/machined (diameter 4 mm and length 8 mm) from rods of commercially pure titanium (grade 4) followed by an anodising process.

#### Surface Roughness

The implant surface roughness was characterized by white light interferometry, GBS, mbH, Ilmenau, Germany and MountainsMap Imaging Topography software (version 7.0, Digital Surf, Besancon, France). Three implants were measured on nine sites each: three tops, three valleys and three flanks. Each measurement had a size of 350 × 224 µm. Errors of form and waviness were removed with a Gaussian filter size of 50 × 50µm. Three parameters were selected to describe the surface, one height descriptive parameter (Sa), one spatial parameter (Sds) and one hybrid parameter (Sdr).

Sa is the average height deviation over the surface calculated from a mean plane. Calculated in µm.

Sds is the density of summits, expressed in number/mm^2^.

Sdr is the increase in surface area compared to a completely flat area reference. Expressed in %.

### 2.2. Animal Model and Surgical Procedure

Ten male Swedish lop-eared rabbits with weight between 3.65 to 4.95 Kg were used in this experiment, with ethical approval (number 188-15) from the Regional Ethics Committee for Animal Research of Malmö/Lund, Sweden. All experiments were carried out in accordance with the rules and regulations of the Swedish Board of Agriculture. The number of the animals included in the study was selected after power analysis performed with G*Power software (version 3.1.9.4, Department of Psychology, University of Düsseldorf, Düsseldorf, Germany) to achieved a statistical power of 80%, given an α-error of 0.05 and an effect size of 0.8.

General anesthesia was induced by intramuscular injection of ketamin (Ketaminol; Intervet, Stockholm, Sweden) and dexmedetomidine (Dexdomitor; Orion Pharma Animal Health, Danderyd, Sweden) followed by subcutaneous injection of buprenorfin (Temgesic; Indivior, Berkshire, Great Britain).

**Figure 1:**
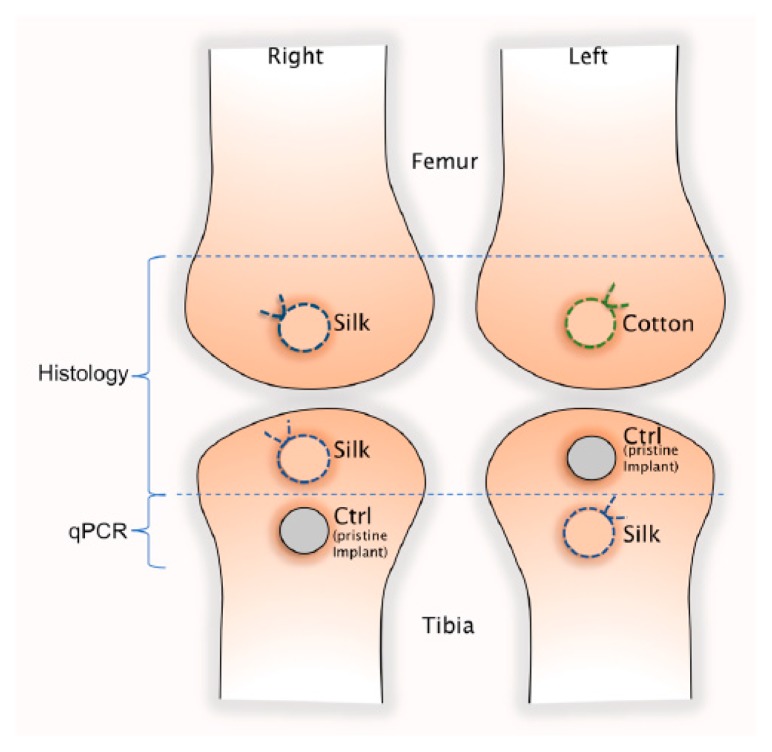
Schematic drawing of implant and ligature placement.

The surgical site was shaved and cleaned with chlorhexidine ethanol solution 0.5 mg/mL (Klorhexidinsprit; Fresenius Kabi, Uppsala, Sweden) and covered with a sterile surgical drape. After injection of lidokain 10 mg/mL (Xylocain; Aspen Nordic, Ballerup, Denmark) at the surgical sites, the femoral and tibial metaphyseal plates were exposed by incision and dissection of covering tissue layers, including skin, muscle and periosteum on the medial side. A total of 60 implants (*n* = 60) were inserted according to Figure 1; one in each condylar metaphyseal plate and two in each tibial metaphyseal plate, with a center to center implant distance of 10 mm.

**Figure 2:**
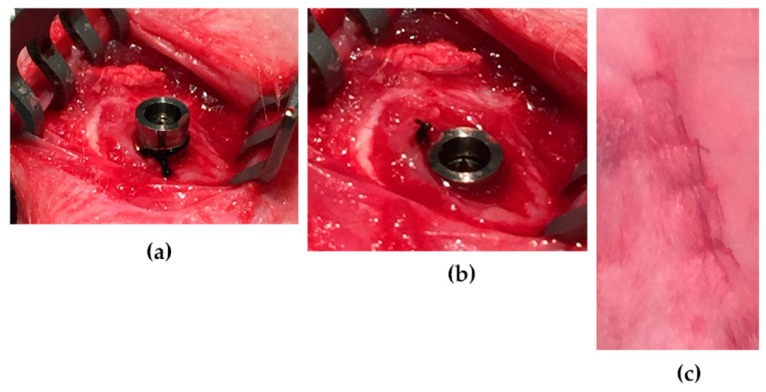
(**a**) Ligature tied around the implant neck after half-way insertion of the implant. (**b**) Ligature compressed against the bone by finishing the implant insertion. (**c**) Wound closure with interrupted resorbable sutures in a layered fashion.

The implant insertion and ligature application technique are depicted in Figure 2. Osteotomies were performed with burrs of increasing diameter up to 3.5 mm under constant irrigation with physiological saline solution. After inserting the implants halfway, a single ligature loop of sterile 3-0 braided silk (Ethicon, Cincinnati, OH, USA) were tied with a surgical knot around the neck of the (i) right femoral, (ii) right proximal and (iii) left distal tibia implants of all rabbits. The ligature was then compressed between the implant neck and marginal bone, by finishing the fixture insertion. With an identical procedure, the left femoral implants were ligated with non-impregnated cotton gingival retraction cord (GingiKNIT non-impregnated; Kerr Dental, Bioggio, Switzerland). The right distal and left proximal implants were inserted without ligatures and used as controls. Multi-layered wound closure was performed with resorbable sutures in fascia and skin (Vicryl 3-0, Ethicon, Cincinatti, OH, USA) at all sites in order to ensure a submerged and non-contaminated healing environment for all implants.

At 8 weeks, the rabbits were sacrificed with a lethal injection of intra peritoneal pentobarbital (Euthasol; Virbac, Kolding, Denmark). Femoral- (*n* = 20) and proximal tibial (*n* = 20) implants were resected en bloc and directly immersed in 4% buffered formaldehyde (Merck, Darmstadt, Germany) for three days.

The distal tibia samples (tissues and implants) were harvested for gene expression analysis in the following way:

(i) The soft tissue that adhered to the implant margin was removed by a 6 mm punch after removing the cutis.
(ii) The implants were then unscrewed and the marginal bone trephined out using a 6 mm trephine.

These respective tissues were placed in RNA-later store in +4 °C overnight and then −20 °C until processing.

### 2.3. Histology

#### 2.3.1. Histological Sample Preparation

Following the formalin fixation, the femoral (*n* = 20) and distal tibia (*n* = 20) samples were rinsed in tap water and then dehydrated in increasing concentrations of ethanol from 70% up to 99.9% (Solveco, Rosersberg, Sweden). The next steps involved pre-infiltration in diluted resin and pure resin followed by embedment in light-curing resin (Technovit 7200 VLC; Heraeus Kultzer, Wehrheim, Germany). All samples were divided in a similar direction i.e., in the center of the implant (in a longitudinal manner of the implant) using the cutting and grinding system, i.e., the ExaktR equipment (Exakt Apparatebau, Norderstedt, Germany). A central section of about 150–200 µm were cut. The samples were ground with Silicon carbide wet grinding papers of 800–1200 grit (Struers ApS, Ballerup, Denmark) to a final thickness of about 15 µm. The section surfaces were cleaned and dried prior to histological staining in toluidine blue mixed with pyronin G. 

Finally, the sections were glass cover-slipped using a routine glue (Pertex, Histolab Products AB, Göteborg). The sample preparation related to the cutting and grinding procedure followed the routine laboratory-guidelines as deduced by Donath et al. [24] and Johansson and Morberg [25,26]

#### 2.3.2. Histological Analysis: Qualitative and Quantitative

The histological sections were qualitatively and quantitatively investigated in a light microscope (L.M., Eclipse Nikon ME600, Nikon, Tokyo, Japan) by two of the authors: C.J. and D.R. Measurements were performed with the NIS-Elements D 64-bit software (version 3.2, Nikon Metrology, SARL, Lisses, France) using a 10× objective. Light microscopic (LM) images of the histological features were obtained with a Nikon DS-Ri1 camera (Nikon Instruments Inc. Meville, NY, USA). The first implant-bone contact was ascertained with a 40× objective.

The distance from the implant top to first bone contact was measured on both sides of each implant (anterior and posterior) sides. The difference between the distances for control versus test (silk ligatures) samples in the proximal tibia and for the cotton versus silk samples in the femur, were analyzed using non-parametric Wilcoxon Signed Rank with the pair considered as the control and test samples from the same rabbit. Statistical significance was set for *p* < 0.05. A level of 0.05 was selected for the α-error and the statistical significance was set for *p* < 0.025 after Bonferroni’s adjustment for multiple calculations. A qualitative investigation of the marginal hard and soft tissues was also performed.

### 2.4. Gene Expression Analysis—qPCR

The samples for the gene expression analysis originated from the soft tissues that were in direct contact with the implant heads in the distal tibias and bone tissue that was adherent to the implants in distal tibia and constituted four study groups (soft tissues specimens from implants with and without ligature and bone specimens from implants with and without ligatures). A total of 32 samples (20 soft tissues and 12 bone tissue) were analyzed. The soft tissues were cut with sterile, disposable tissue punches of 6 mm diameter (1 punch per specimen) and dissected from the implant surface with a curette (Miltex, Inc. York, PA, USA). 

The specimens were immediately placed in separate sterile plastic tubes containing RNA*later* solution (Ambion, Inc., Austin, TX, USA), for fixation. The samples were then stored at 4 °C overnight and further stored at −20 °C until processing.

#### 2.4.1. mRNA Isolation

(This step is performed to purify the mRNA from the samples.)

mRNA isolation and qPCR amplification were performed at TATAA Biocenter, Gothenburg, Sweden.

Before mRNA isolation and extraction, the samples were thawed on ice. The samples were extracted using Qiazol (Cat.No 79306) and the RNeasy mini kit (Cat.No 74104) (Qiagen GmbH, Venlo, Netherlands) according to manufacturer’s instructions. 

Sample concentrations where determined using spectrophotometry (Dropsense, Trinean, Pleasanton, CA, USA) and RNA integrity was analyzed using capillary electrophoresis (Fragment Analyzer, Thermo Fisher Scientific, Waltham, MA, USA). 

After extraction, the RNA was cleaned of inhibitory factors using the RNeasy MinElute Clean up kit (Qiagen GmbH, Venlo, Netherlands, Cat no. 74204). 

#### 2.4.2. Reverse Transcription (RT) 

(This step produces complementary DNA (cDNA) to the mRNA isolated from the respective samples.)

RNA was reverse transcribed in single 20 µL reactions on all 32 samples. 

Samples where first normalized to 33.33 ng/µL to reach a quantity of 500 ng RNA per tube that was loaded into the reaction. The RT was performed according to manufacturer’s instructions with TATAA Grandscript cDNA synthesis kit Cat.No A103 (TATAA Biocenter AB, Gothenburg, Sweden), with the following concentration: 1 µL RT enzyme, 4 µL reaction mix, 15 µL normalized sample RNA. 

#### 2.4.3. Assays Design and Validation

(This step is performed to create short DNA fragments that are later used to determine each specific region of DNA to be copied, in this case the regions that encode the proteins of interest for the study.)

Eleven assays where designed and validated by TATAA (according to SOP 009 ver 1.2 SOP 001 ver1.4) (Validation plan qPCR assay validation). A list of all assays can be found in Table 1. For validation, a control genomic DNA (gDNA) from male rabbit (Zyagen) and a pool of all the cDNA samples was used. Seven-point standard curves in 10-fold dilutions were run for all the assays, in quadruplicates. The annealing temperature was set to 60 °C for all assays. PCR products; gDNA, cDNA, selected standard point and NTC were then run on Fragment Analyzer using the DNF-910-33-DNA 35-1500bp kit (Thermo Fisher Scientific, Waltham, MA, USA) to check the product lengths and primer specificity. The qPCR data for the assay validation was analysed with GenEx software (version 6, MultiD Analyses AB, Göteborg, Sweden) to calculate the efficiencies and performance of the assays with a confidence interval of 95%.

#### 2.4.4. Real-Time Quantitative Polymerise Chain Reaction (RT-qPCR)

(This final step monitors the amplification of the selected DNA segments in real time and enables the researcher to quantify the amount of each DNA segment at the start of the reaction.)

The cDNA samples were diluted 10× to have enough volume and were then analyzed using TATAA SYBR GrandMaster^®^ Mix Cat. No. TA01-625 (TATAA Biocenter AB, Gothenburg, Sweden). Five µl of TATAA SYBR Green Master Mix, 0.4 µL of Primer (forward & reverse), 2.6 µL of nuclease-free water and 2 µL of cDNA templates were used for each reaction mix. All pipetting was performed by a pipetting robot (EpMotion 5070, Eppendorf, Germany). Duplicate NTCs were included for all the assays and cDNA samples were run in duplicate reactions. Universal ValidPrime assay, Cat No. A107P (TATAA Biocenter AB, Göteborg, Sweden) was used to compensate for possible gDNA contamination.

The quantification was performed using the LightCycler 480 (Roche, Basel, Switzerland) and detection was performed in the SYBR channel. Cq values were based on the second derivative maximum threshold method. Inter-plate calibrator, IPC Cat. No. IPC250 (TATAA Biocenter AB, Göteborg, Sweden) was run on each plate to be able to correct for inter-run differences. qPCR raw data were pre-processed and analyses with GenEx software (version 6, MultiD Analyses AB, Gothenburg, Sweden) and were thereafter imported in Qbase+ software (version 3.1, Biogazelle, Zwijnaarde, Belgium) for calibrated normalized relative quantification of the gene expression. Three assays were used as reference genes (ACTB, GAPDH and LDHA) and their quality as reference genes was assessed with the GeNorm algorithm.

#### 2.4.5. Statistical Analysis

The gene expression results were reported as calibrated normalized relative quantities (CNRQ). Mean and 95% confidence intervals were reported for each assay for each group.

The difference in mean between the test (silk ligated implants) and the control (pristine Ti-implant) groups in the soft tissue samples and between the test and control groups in the bone samples were analyzed using non-parametric Wilcoxon Signed Rank. The test and control samples from the same rabbit were considered as paired. The level of *α*-error was set to 0.05 and statistical significance was adjusted according to Bonferroni´s method for multiple testing; therefore, it was set to *p* < 0.0027.

## 3. Results

### 3.1. Clinical Appearance at Sacrifice

**Figure 3:**
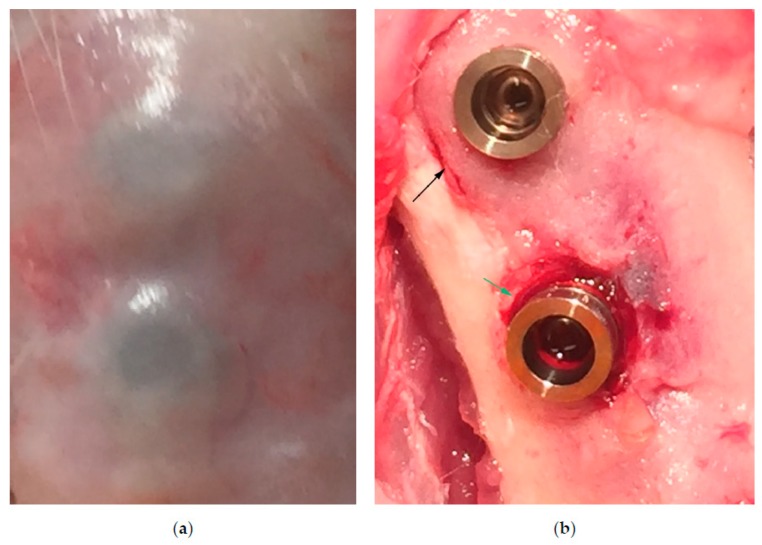
(**a**) The soft tissue covering a tibial control implant (superior) and test implant (inferior) after removal of skin and subcutaneous tissues. (**b**) A small saucerization-like defect (green arrow) visible after removal of the silk ligature around the inferior implant and callus formation (black arrow) around the superior control implant.

The tissue harvesting procedure is shown in Figure 3. Uneventful healing and primary wound closure were achieved in all cases. Dissection of the tissues revealed no pus or other signs of infection around the implants. Exposure of the bone adjacent to the implants revealed small saucerization-like defects around most ligated implants and sometimes callus formation lateral to the ligatures. Some control implants presented with callus formation lateral to the implants, but no saucerization-like bone defects were noted.

### 3.2. Histological Results

#### 3.2.1. Histomorphometrical Results

**Figure 4:**
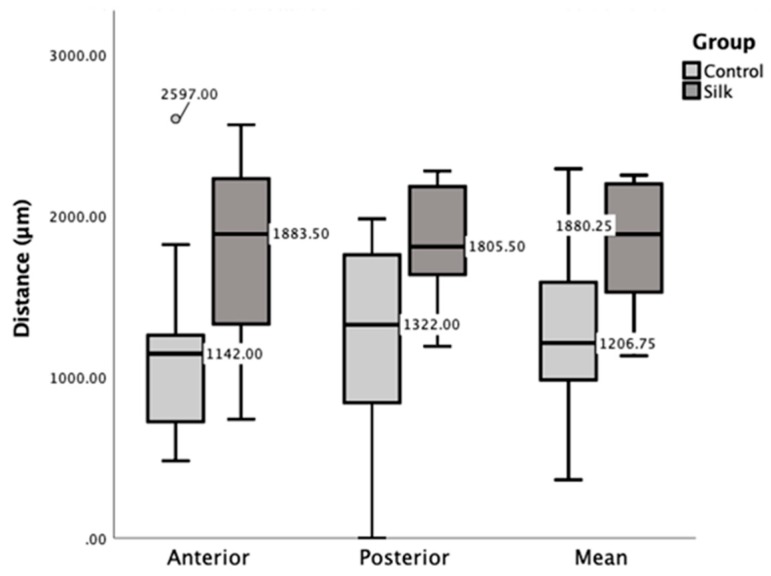
Box-plot showing the distance from implant top to first bone-to-implant contact for implants with silk ligatures (Silk) and pristine implants (Control).

One implant with a cotton ligature was excluded from the histomorphometrical analysis due to a superior displacement of the ligature and unfavorable implant location that engaged the dorsal femoral bone plate and showed non-union of the implant on that side. The distance from the implant top to first bone contact was significantly longer for test implants ligated with silk compared to controls (*p* = 0.007) (Figure 4). This difference was due to the combined effect of bone resorption inferior to the ligatures in the test implants and also bone gain due to callus formation adjacent to protrusive implant tops in some control implants. The difference between implants ligated with silk or cotton was not statistically significant (*p* = 0.37).

#### 3.2.2. Qualitative Histological Results

##### Control Implants (No Ligature Involved) 

**Figure 5:**
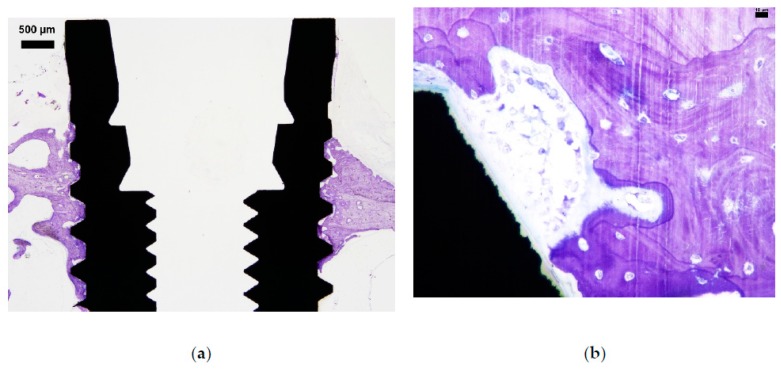
(**a**) A typical example of a control sample (no ligature involved) with darker stained periosteal and endosteal new bone formation. (**b**) This figure illustrates seemingly an ongoing bone remodeling region with clear demarcation between old bone (lighter stained) and younger bone (larger osteocytes and a bit darker stained bone tissue). Possibly some macrophages can be observed in the region closer to the implant.

Control samples are shown in Figure 5. The control samples demonstrated both periosteal and endosteal new bone formation. The old cortical bone surfaces, observed in regions close to the implant, were undergoing remodeling with bone forming and bone resorption areas present. Most control sections demonstrated that the implant interface regions consisted of new formed bone with various amounts of bone-to-implant contact (BIC) and active bone forming regions (bone tissue covered by osteoid with osteoblasts visible) close to the implant. In some sections, multinucleated giant cells (MNGCs) of various sizes could be observed in close vicinity to metal oxide debris and particles (most likely detached from the bulk implant being anodized).

##### Test Implants (Ligated with Silk or Cotton)

**Figure 6:**
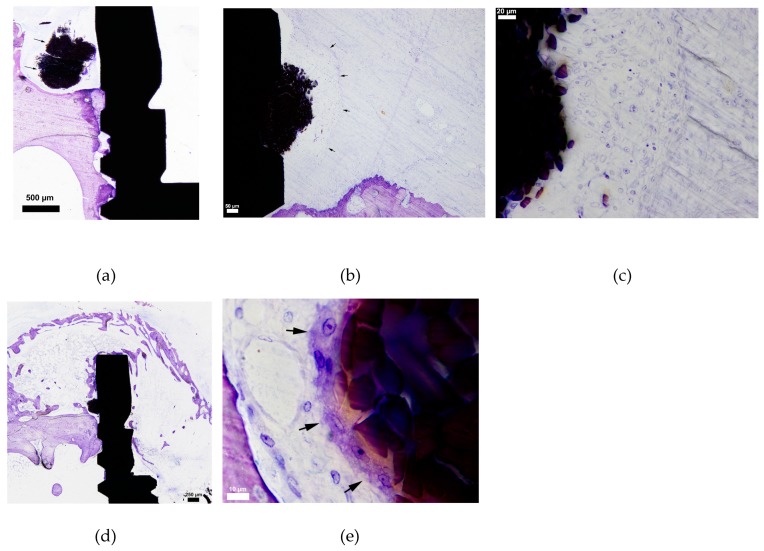
Sample figures from different silk sections. (**a**) Survey figure of a typical section with a silk ligature (arrows) above a partly resorbed cortical bone surface in the periosteal region. (**b**) In some cases, the silk ligatures (in close contact to the implant) were surrounded by a thick cellular infiltrate layer (arrows) dominated by macrophages of various sizes and shapes. Outside this formation, loose connective tissue was formed. (**c**) The same section in higher resolution. (**d**) This figure illustrates a rather large “dome-like” callus formation of the periosteal bone and it seems like the implant part above the silk ligature is almost covered by new formed bone. (**e**) The arrows illustrate a large, elongated multinucleated giant cell (MNGC) in intimate contact with the silk.

Silk samples are shown in Figure 6. The silk samples demonstrated soft tissue encapsulation of the material with capsules of varying thickness and sometimes also distant bony encapsulation. The capsules appeared both as a “loose” and a “tight” formation with macrophages of various sizes as the dominating cell type. Possibly some plasma-cells were also part of the cell population in such regions. One section demonstrated loosened single silk fibers in the soft tissue region appearing as being encapsulated by a rather thick formation of macrophages of various sizes and shapes. The silk material was not in contact with bone and a capsule formation separating bone and silk could sometimes be observed. In five samples, MNGCs could be seen as long elongated rims that captured the silk material (i.e., foreign body reaction). This rim of cells was involved in a soft tissue space that separated the bone from the silk. The bone surfaces, at some distance away, showed signs of resorption but no osteoclasts could be observed. Macrophages of various sizes and shapes were observed in the soft tissue regions close to the implant surface. The silk material was most often located “above” the bone surface, seemingly glued onto the implant, compared to cotton being “spread out”. One silk sample demonstrated a shell/dome-like bone formation with marrow tissue at the periosteal side. 

**Figure 7:**
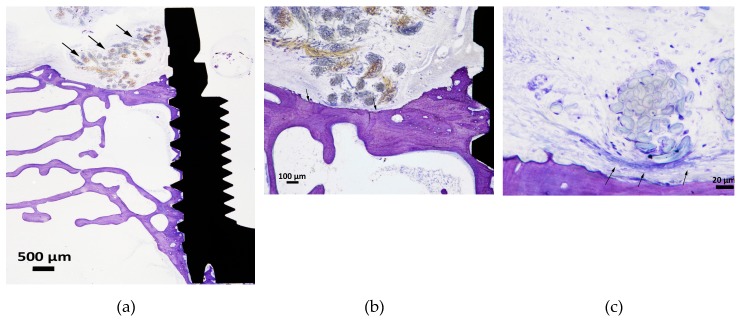
Three images from one cotton section with arrows showing the cotton ligature. (**a**) An illustration of a typical section with cotton suture situated above the periosteal bone. (**b**) The periosteal bone surface is separated from the cotton ligature by a soft tissue layer and the bone surface appears to be resorbed (arrows), although no osteoclasts could be observed. (**c**) The amount of macrophages in the soft tissue between the bone and the cotton seemed to be less compared to the silk sections. However, macrophages were visible as being more spread out and “darker” compared to silk samples. None of the cotton samples demonstrated a typical MNGC formation and the “encapsulation” of cotton was often loosely arranged (arrows).

Cotton samples are shown in Figure 7. The majority of the sections of the cotton material could be observed as “huge loosened regions” with the material encapsulated by soft tissue situated above the periosteal bone surface. The capsule itself was often surrounded by bone trabecula and the interface between the periosteal bone surface and soft tissue seemed to undergo resorption, with regions of “mouse-eaten” bone; however, no osteoclasts could be observed. Cotton demonstrated a larger diameter of the material than silk, with several separate “cotton rolls” visible both at a distance from the implant and in close contact, compared to the silk. No active bone forming surface (i.e., osteoid rim with osteoblasts) could be observed close to the soft tissue. Although the bone surface, in general, seemed to be resorbed (“mouse-eaten” surface) no osteoclasts were visible, except in one cotton-section. In higher magnification MNGCs could be observed in close vicinity to cotton. Macrophages were also visible but seemingly in less amount (albeit not counted) compared to silk. Cotton seemed to have a greater soft tissue area surrounding the material compared to silk, which possibly indicated a higher degree of encapsulation of cotton compared to silk.

### 3.3. Gene Expression Results

**Figure 8:**
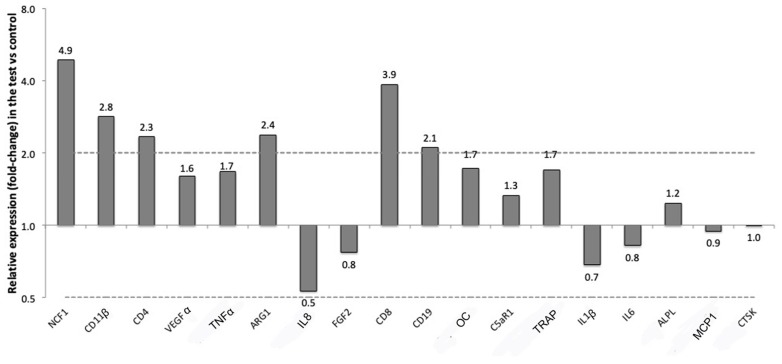
Up and down regulation of the selected markers in the soft tissues around implants with silk ligatures versus controls (no ligature).

**Figure 9:**
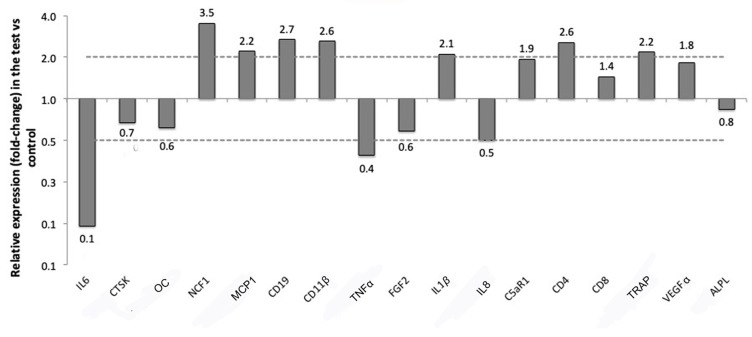
Up and down regulation of the selected markers in the bone around implants with silk ligatures versus controls (no ligature).

The expression of a panel of genes in the soft tissue around titanium implants either left pristine (control) or treated with a silk ligature placed around the implant neck (test) was evaluated with RT-qPCR. The relative expressions of the selected markers are presented in Table 2 and Figure 8 for soft tissue and in Table 3 and Figure 9 for bone tissue.

In the soft tissues near the silk ligature, several genes mediating reactions of immune cells were more than two-folds up-regulated compared to the controls. Those were NCF1 (CNRQ 4.9), which is specific for neutrophils, CD8 (CNRQ 3.9), which is a marker for T-lymphocytes, CD11β (CNRQ 2.8), a M1 macrophages marker, ARG1 (CNRQ 2.4), a marker for M2 macrophages, CD4 (CNRQ 2.3), which is another gene specific for T-lymphocytes, and CD19 (CNRQ 2.1), which is associated with B-lymphocytes. One gene, IL8, related to macrophages was two-folds down-regulated in the tests versus the controls. None of the markers reached a level of significance of *p* ≤ 0.0025 in expression between the tests and the controls, which was the adjusted *p*-value (Table 2, Figure 8).

In the bone surrounding the implants ligated with silk, six genes related to the immune response were expressed more than two-folds compared to the bone surrounding pristine implants. Of those, four were the same that were overexpressed in the soft tissues near the ligatures: NCF1 (CNRQ 3.5), CD19 (CNRQ 2.7), CD11β (CNRQ 2.6) and CD4 (CNRQ 2.6). Other genes up-regulated in the bone of the test samples were MCP1 (CNRQ 2.2), which is related to macrophage fusion, ILβ1 (CNRQ 2.1) which is another gene related to M1 macrophages, and Triiodothyronine receptor auxiliary protein (TRAP) (CNRQ 2.2), which is an osteoclast marker for bone resorption.

Three genes were instead two-folds or more down-regulated in the bone around the test implants compared to the controls. They were IL6 (CNRQ 0.1), which is a cytokine related to inflammation, but also to bone formation [1], TNFα (CNRQ 0.4), another cytokine related to inflammation, and IL8 (CNRQ 0.5), which is related to macrophages and that was down-regulated also in the soft tissues of the test implants. None of these investigated genes in the bone reached a level of significant difference in expression for *p* ≤ 0.0025 (Table 3, Figure 9).

## 4. Discussion

The current study described marginal peri-implant bone and soft tissue reactions to marginal silk and cotton ligatures without plaque accumulation. Our findings present clear criticism to the present interpretation of ligature models that they verify bacteria to be the initiating problem behind marginal bone loss. In reality, previous claims that ligatures induce bone loss solely by plaque accumulation and not by themselves are incorrect [4,18,26]. The present findings also raise questions as to what extent potential clinical provocations may also induce bone loss. Consider for example apically displaced cementum residues, unsuitable, lose or ill-fitting abutments or increasing amounts of wear debris in peri-implant tissues over time. Indeed, wear particles of both cementum and titanium has been found in abundance in human biopsies of peri-implantitis lesions [27]. Furthermore, the possible role of sterile Ti debris in the development of soft tissue inflammation and marginal bone resorption was recently demonstrated by Wang et al., who showed marginal bone resorption around submerged titanium implants in Sprauge Dawley rats and also demonstrated a role of M1 macrophages in that process. [28]. Future studies may strive to provide more knowledge about potential aseptic causes for marginal bone resorption, with the ultimate goal to prevent and treat such conditions. 

As described by Donath et al., the body will always strive to alienate an implanted material by rejection, dissolution, resorption, demarcation (i.e., fibrous or bony encapsulation) or a combination of these reactions [29]. The type and extent of the immunological response has been shown to depend on multiple factors, such as material type, surface characteristics, type of receiving tissue, degree of surgical trauma, micromovement between material and host and other factors [7,29]. From studies on osteoimmunology we know that focal bone loss is achieved by osteoclasts when activated by adjacent inflammation, and that TNFα is likely the most significant inflammatory cytokine necessary to activate the osteoclasts [30]. TNFα is expressed in the acute inflammatory response against many types of provocations, such as surgical trauma or presence of infectious microorganisms, necrotic tissue or certain foreign materials. Hence, bone resorption can be expected to occur from almost any type of adjacent, pro-inflammatory provocation of a certain magnitude and may also be continuous if the provocation is sustained or repeated. In experimental peri-implantitis, this is well-exemplified by the rapid marginal bone loss that often occurs when ligatures are frequently exchanged and new ligatures are pushed apically towards the bone, as compared to the frequent self-containing resorption that often occurs from non-exchanged ligatures [17].

The extensive, macrophage-dominated infiltrates adjacent to the silk and cotton ligatures in the present study demonstrates a stronger inflammatory reaction to the ligatures compared to non-ligated control implants, which may likely explain the resorbed bone defects frequently found adjacent to the ligatures. Macrophages can initiate bone resorption in different ways: indirectly by secretion of pro-inflammatory cytokines that stimulate osteoclast generation and activation as described above [30] or directly by the secretion of certain matrix metalloproteinases that can degrade bone matrix [31]. However, the present study demonstrated a late stage foreign body reaction, with fibrous encapsulation of the ligatures, lack of up-regulation of the pro-inflammatory cytokine markers for TNFα, IL1β and IL6 in the soft tissues around them and seemingly arrested bone resorption as evident by the lack of osteoclasts in the resorbed bone defects adjacent to them. This late stage reaction was likely due to the long healing time of 8 weeks and is consistent with the previous findings of Setzen et al., who reported a quite extensive inflammatory response to black braided silk sutures during the first few weeks, followed by the thickest fibrous capsule formation compared to 10 other suture types inserted subcutaneously in rabbits [32]. Recent findings by Nguyen et al. suggested that active bone resorption occurs much earlier, even when plaque accumulation is allowed. In their recent experimental peri-implantitis study in mice, high expression levels of TNFα and IL1 were observed during the first 2 weeks after application of plaque accumulating 5-0 silk-ligatures, followed by a subsequent decrease back to baseline levels after 4 weeks. Simultaneously, the number of osteoclasts and rate of marginal bone resorption both decreased towards the end of the study at 4 weeks [23]. The tissue reactions to strictly bacterial assaults also share many similarities, and is perhaps best exemplified by the closed confines of the periapical bone, when provoked by endodontic pathogens. As explained by Nair et al., the endodontic pathogens typically trigger an acute inflammatory reaction with simultaneous active bone resorption, followed by a chronic stage, or equilibrium, with arrested bone resorption and a dense fibrous capsule that shields off the inflammatory infiltrate from the bone while it continues to hold back the infection [33]. New acute episodes may then be triggered years later, in response to changed local or systemic circumstances. 

In contrast to the complex combined assault of repeated mechanic tissue trauma, hostile ligatures and hostile bacteria provided by classical experimental peri-implantitis, the present study demonstrated the isolated capacity of silk and cotton ligature to initiate bone loss. While the distance from the implant top to the first bone contact point varied significantly between silk and controls, the difference between silk and cotton was not significant. However, the fact that the soft tissue capsules that separated the ligatures from the bone were thicker around cotton than silk, may indicate a more consistent bone resorption against cotton. Future studies could benefit from a histomorphometrical method, which unfortunately cannot be performed successfully on non-decalcified, resin-embedded, cut-and-ground sections. The difference in bone loss between similar study subjects (the rabbits in our study) conforms to the findings of traditional ligature induced peri-implantitis studies, where the time to achieve a certain amount of bone loss has also varied greatly [34,35]. These differences are likely due to both immunological differences between animals, as indicated by recent knock out models [36], as well as methodological aspects such as possible variations in the distance between ligature and bone at the time of ligation. The choice of a rabbit model in the present study makes it difficult to compare the present results to those of previous studies that used different mammals as experimental models, considering the great differences in the resulting bone loss for different animal species [17]. For example, Beagle dogs have shown >3 mm of bone loss after 10-weeks with ligatures [37], while monkeys have sometimes required a year of regularly exchanged ligatures to induce 1 mm of bone loss [15].

Another important difference between the present and previous studies was the simultaneous implant and ligature insertion, as opposed to the few months of implant healing before ligation utilized in the majority of previous studies. Jovanovic et al. demonstrated that the amount of marginal bone loss and configuration of peri-implant pockets did not differ from ligation at implant placement compared to a preceding 3 months implant healing time [38].

Although the impact of the ligature has often been overlooked in ligature induced peri-implantitis studies, as evident from the fact that some authors have not specified any details about the type of material used [39,40], information about the ligature materials can be found from other research fields. The most commonly used ones are the organic materials cotton and silk. In surgery, cotton gauze sponges are used to soak up fluids and maintain the surgical field, but are then carefully removed from the tissues due to their capacity to provoke extensive foreign body reactions. When extensive, these foreign body reactions can manifest themselves as tumor-like lesions referred to as cotton-ballomas or gossypibomas. In some cases, these lesions have induced significant bone resorption and may then mimic an osteolytic tumour such as a sarcoma [41,42]. A study on mice also showed that even microscopic remnants of sterile cotton may induce foreign body reactions when left in a surgical wound [43].

Silk, on the other hand, has been used as a suture material for over a century, and the black, braided silk sutures used in the present study are made from fibroin, extracted from virgin Bombyx mori silk in a process that separates the fibroin from a second, glue-like protein called sericin [44]. In addition to fibroin, the utilized suture type also contains a beeswax coating. It is unknown to what extent this particular coating affects the immunological response, but it should be noted that beeswax is the main ingredient of bone wax, a product used to stop osseous bleeding during surgery and well known to arrest bone healing and induce significant foreign body reactions [45]. Older pre-1980s silk sutures contained both fibroin and sericin and were known to induce considerable acute and chronic inflammatory responses, as well as frequent late allergic reactions. Modern fibroin type silk sutures very rarely induce allergy, but still elicit a relatively strong acute inflammatory reaction in the early healing phase. Silk also undergoes slow proteolytic degradation, even though the sutures are defined as non-resorbable [44,46]. Spelzini et al., compared two types of fibroin silk implants with a polypropylene implant for fascial repair in mice and reported a somewhat stronger acute inflammatory reaction to the silk implants, followed by a much stronger chronic inflammatory response with progressive accumulation of chronic inflammatory cells up until 30 days that remained virtually unchanged after 90 days. They also reported a high initial presence of polymorphnuclear neutrophil (PMN) cells, which subsequently dropped in numbers with time but still remained in smaller numbers after 90 days [46].

The clinical significance of the PCR results of the present study must be considered in light of the histological sections and the evident late healing stage and arrested bone resorption described above. It must also be kept in mind that the PCR results only demonstrate the difference between test (silk ligature) and control (no ligature) implants, and hence do not show the immunological reaction to the Ti-implants that were identical for tests and controls. For example, CD11β was 2.8 times upregulated in the present study but 13 times upregulated in response to Ti compared to sham after 28 days in a previous rabbit study, which suggests a pronounced immunological reaction dominated by macrophages to Ti that was masked in the present study design [47]. Additional biopsies at baseline or from untouched distant tissues at sacrifice may facilitate the interpretation in future studies. Regarding the bone specimens, the small differences between tests and controls may in part be due to the harvesting technique, considering that the entire implants + surrounding bone were harvested and analyzed, while only a very small marginal portion of them was ever in contact with the ligatures.

With the above factors in mind, the more than two-fold upregulation of the soft tissue markers NCF1, CD11β, and CD4, ARG1, CD8 and CD19 for silk ligated test implants compared to pristine implants demonstrated a greater activation of the immune response in the test compared to the controls that corresponded to the chronic inflammatory cell infiltrates present around the ligatures. The upregulation of CD11β and ARG1 indicate a mixed M1/M2 phenotype of the macrophages in this tissue. CD4 and CD8 upregulation indicate T-cell presence. T-cells play a key role in antigen specific defense, but are also involved in foreign body reactions in absence of known antigens [48]. Their increased presence in the present study may correspond to the larger number of adherent macrophages and MNGCs on the silk ligatures as compared to controls (Ti), as demonstrated by Brodbeck et al., who described that lymphocytes (mainly CD8+ T-cells and CD4+ T-cells) “rosetted around” biomaterial-adherent macrophages and MNGCs in a co-cell culture study [48]. The authors further demonstrated that the presence of lymphocytes augmented macrophage adherence to biomaterials as well as MNGC-fusion, when both cell types were present from the start of the in vitro experiment [48]. However, a later study on T-cell deficient mice demonstrated a seemingly normal foreign body response with adhesion and fusion of macrophages to an implanted material even in absence of T-cells, which indicates that, while present, T-cells are probably not necessary for a normal foreign body reaction to occur [49]. The prolonged neutrophil presence in soft tissue indicated by upregulation of NCF1 further indicates a more pronounced foreign body reaction to the silk ligatures than controls (no ligature involved). Recent studies demonstrate a long-term role of neutrophils in foreign body reactions, as well as a capacity for them to regulate the long term reaction toward an implant [50]. Jhunjhunwala et al., recently demonstrated a 30-500-fold increased neutrophil presence in the peritoneal lavage of mice in response to sterile implanted microcapsules after 2 weeks, which is much longer than the hours or few days they have previously been thought to survive at a wound site. Jhunjhunwala et al. further demonstrated that the neutrophils became activated in response to the implant, resulting in the secretion of different immunomodulatory cytokines and chemokines and formation of extracellular traps (NETs) on the material surface [51]. The increasing knowledge about the pivotal role of neutrophils in the regulation of foreign body reactions suggests that a strong acute inflammation, associated with the implantation, can predispose an equally strong chronic inflammation orchestrated by neutrophils and characterized by prolonged neutrophil presence and frustrated phagocytosis in the very long run [50]. Beside the impact of material properties on the long-term neutrophil response indicated in the present study, the eventual influence of other factors, such as traumatic surgery and pre-existing disorders that influence the inflammatory response, such as diabetes, may be considered in future studies [52,53,54]. 

The immunological reactions to aseptic provocations of dental implants remains largely unexplored and future studies may focus on investigating the details in the immune response to different provocations at different time points throughout the healing phase, as well as refining the methods for such investigation.

## 5. Conclusions

Within the limitations of the present study, it was demonstrated that aseptic marginal ligatures made of silk and cotton triggered an immunological reaction in the peri-implant tissue of rabbits, with abundant numbers of inflammatory cells in contact with the ligatures. Marginal bone resorption was also evident adjacent to the ligatures, which rejected our null-hypothesis that ligatures cannot induce bone resorption in the absence of a bacterial plaque. Future studies may aim to describe the tissue reactions at both earlier and later time points, as well as further elucidate the details of the immunological events responsible for bone resorption adjacent to aseptic marginal ligatures.

## Figures and Tables

**Table 1:** Gene sequences and biological entity.

Primer	Forward Sequence	Reverse Sequence	Accession Number	Biological Entity
ACTβ	GAGATGCCATGTGACGGAAG	TTACACAAATGCGATGCTGC	NM_001101683.1	Reference gene
ALPL	ACTGTGGACTACCTCTTG	GGTCAGTGATGTTGTTCC	XM_017346489	Bone mineralization
ARG1	GGATCATTGGAGCCCCTTTCTC	TCAAGCAGACCAGCCTTTCTC	NM_001082108.1	M2 macrophage
C5aR1	ACGTCAACTGCTGCATCAACC	AGGCTGGGGAGAGACTTGC	NM_017338812.1	The complement system
CD11β	TTCAACCTGGAGACTGAGAACAC	TCAAACTGGACCACGCTCTG	XM_008248697.2	M1 macrophage
CD19	GGATGTATGTCTGTCGCCGT	AAGCAAAGCCACAACTGGAA	NM_002711879.3	B lymphocytes
CD4	CAACTGGAAACATGCGAACCA	TTGATGACCAGGGGGAAAGA	NM_008254148.2	T lymphocytes
CD8	GGCGTCTACTTCTGCATGACC	GAACCGGCACACTCTCTTCT	NM_008254148.2	T lymphocytes
GAPDH	CCGAGACACGATGGTGAAGG	TGTAGACCATGTAGTGGAGGTCA	NM_001082253.1	Reference gene
IL8	CTTTTTGCCCTGACCATGCC	TCCTTCACAAGCGAGACCAC	NM_001171082.1	Macrophage
LDHA	ACAAGTGCACAAACAAGTGGT	AGAGCCCCTTAAGCATGGTG	NM_001082277	Reference gene
MCP1	GCTCATAGCAGTCGCCTTCA	CATGAAGATCACAGCTTCTTTGGG	NM_001082294	Macrophage fusion
NCF1	TTCATCCGCCACATTGCCC	GTCCTGCCACTTCACCAAGA	XM_001082102.1	Neutrophil
OC	AGAGTCTGGCAGAGGCTCAG	TCGCTTCACCACCTCGCT	XM_002715383	Bone mineralization
CTSK	ACTCTGAAGATGCCTACCCCT	TTCAGGGCTTTCTCATTCCCC	NM_001082641	Bone resorption
FGF2	ATCTACACTGTGGAGCTTGCAG	TCATGCGGTCACACACTTCC	XM_002711077	Fibroblast
IL1β	TCCTTGGTGTTGTCTGGCAC	GGCCACAGGTATCTTGTCGTT	NM_001082201	M1 macrophage
IL6	GAGGAAAGAGATGTGTGACCAT	AGCATCCGTCTTCTTCTATCAG	NM_001082064	M1 macrophage
VEGFα	CTTGGGTGCATTGGAGCCTT	CTTCACCACTTCGTGGGGTTTA	XM_017345155	Endothelial cells
TNFα	CTCTTTCCTGCTCGTGGCTG	GGAGGTTGTTTGGGGACTCTT	NM_001082263	M1 Macrophage
TRAP	CTGGGTTTGCAGGAGTTG	TTGAAGAGCAGCGACAGA	NM_001081988	Bone resorption

ACTβ, β-actin (reference gene); ALPL, alkaline phosphatase; ARG1, arginase 1; C5aR1, complement C5a Receptor 1; CD11β, macrophage marker CD11β; CD19, B-lymphocyte surface protein CD19; CD4, T-cell surface glycoprotein CD4; CD8, T-cell transmembrane glycoprotein CD8; GAPDH, glyceraldehyde 3-phosphate dehydrogenase (reference gene); IL8, interleukin 8 receptor alpha; LDHA, lactate dehydrogenase A (reference gene); MCP1, monocyte chemotactic and activating factor; NCF1, neutrophil cytosolic factor; OC, osteocalcin; CTSK, Cathepsin K; FGF2, fibroblast growth factor 2; IL1β, interleukin 1 beta; IL6, interleukin 6; VEGFα, vascular endothelial growth factor alpha; TNFα, tumor necrosis factor alpha; TRAP, Triiodothyronine receptor auxiliary protein.

**Table 2:** Relative expression of the selected gene targets in the soft tissues around implants with silk ligature versus the soft tissues around controls (no ligature involved).

Target Gene	Relative Expression in Test versus Control	95% CI Low	95% CI High	*p*-Value
NCF1	4.9	1.9	12.4	0.008
CD11β	2.8	1.5	5.4	0.016
CD4	2.3	1.7	3.1	0.016
VEGFα	1.6	1.0	2.5	0.05
TNFα	1.7	0.9	2.9	0.08
ARG1	2.4	0.9	6.5	0.11
IL8	0.5	0.2	1.2	0.11
FGF2	0.8	0.5	1.2	0.11
CD8	3.9	0.5	27.4	0.22
CD19	2.1	0.5	12.3	0.22
OC	1.7	0.7	4.2	0.25
C5aR1	1.3	0.9	2.0	0.31
TRAP	1.7	0.5	5.4	0.37
IL1β	0.7	0.2	2.5	0.58
IL6	0.8	0.3	2.5	0.58
ALPL	1.2	0.5	3.1	0.64
MCP1	0.9	0.3	2.8	0.84
CTSK	1.0	0.7	1.5	0.84

CI = confidence interval. *p*-value was calculated with Wilcoxon Signed Rank test, significance level was set to *p* ≤ 0.0027 after Bonferroni´s adjustment for multiple testing. No gene showed significant difference in expression.

**Table 3:** Relative expression of the selected gene targets in the bone around implants with silk ligature versus the bone around controls (no ligature).

Target Gene	Relative Expression in Test versus Control	95% CI Low	95% CI High	*p*-Value
IL6	0.1	0.0	0.9	0.04
CTSK	0.7	0.5	1.0	0.04
OC	0.6	0.4	1.1	0.07
NCF1	3.5	0.8	15.2	0.07
MCP1	2.2	0.8	5.9	0.08
CD19	2.7	0.6	12.3	0.13
CD11β	2.6	0.6	12.2	0.14
TNFα	0.4	0.1	2.3	0.19
FGF2	0.6	0.2	1.6	0.19
IL1β	2.1	0.5	8.8	0.19
IL8	0.5	0.1	2.4	0.26
C5aR1	1.9	0.4	9.3	0.28
CD4	2.6	0.3	25.0	0.28
CD8	1.4	0.4	5.2	0.42
TRAP	2.2	0.1	40.0	0.46
VEGFα	1.8	0.2	20.4	0.49
ALPL	0.8	0.2	4.4	0.76

CI = confidence interval. *p*-value calculated with Wilcoxon Signed Rank test, significance level was set to *p* ≤ 0.0027 after Bonferroni´s adjustment for multiple testing. No gene showed significant difference in expression.

## References

[1] Moraschini V., Poubel L.A., Ferreira V.F., Barboza Edos S. (2015). Evaluation of survival and success rates of dental implants reported in longitudinal studies with a follow-up period of at least 10 years: A systematic review. Int. J. Oral Maxillofac Surg..

[2] Doornewaard R., Christiaens V., De Bruyn H., Jacobsson M., Cosyn J., Vervaeke S., Jacquet W. (2017). Long-Term Effect of Surface Roughness and Patients’ Factors on Crestal Bone Loss at Dental Implants. A Systematic Review and Meta-Analysis. Clin. Implant. Dent. Relat. Res..

[3] Albrektsson T., Buser D., Sennerby L. (2012). Crestal bone loss and oral implants. Clin. Implant. Dent. Relat Res..

[4] Lindhe J., Berglundh T., Ericsson I., Liljenberg B., Marinello C. (1992). Experimental breakdown of peri-implant and periodontal tissues. A study in the beagle dog. Clin. oral implant res..

[5] Zitzmann N.U., Berglundh T. (2008). Definition and prevalence of peri-implant diseases. J. Clin. Periodontol..

[6] Albrektsson T., Dahlin C., Jemt T., Sennerby L., Turri A., Wennerberg A. (2014). Is marginal bone loss around oral implants the result of a provoked foreign body reaction?. Clin. Implant Dent. Relat. Res..

[7] Albrektsson T., Chrcanovic B., Ostman P.O., Sennerby L. (2017). Initial and long-term crestal bone responses to modern dental implants. Periodontology 2000.

[8] Frydman A., Simonian K. (2014). Review of models for titanium as a foreign body. C.D.A. J..

[9] Jemt T., Sunden Pikner S., Grondahl K. (2015). Changes of Marginal Bone Level in Patients with “Progressive Bone Loss” at Branemark System(R) Implants: A Radiographic Follow-Up Study over an Average of 9 Years. Clin. Implant Dent. Relat. Res..

[10] Albrektsson T., Becker W., Coli P., Jemt T., Molne J., Sennerby L. (2019). Bone loss around oral and orthopedic implants: An immunologically based condition. Clin. Implant Dent. Relat. Res..

[11] Harris W. (2018). Vanishing Bone—Conquering a Stealth Disease Caused by Total Hip Replacements.

[12] Landgraeber S., Jäger M., Jacobs J.J., Hallab N.J. (2014). The pathology of orthopedic implant failure is mediated by innate immune system cytokines. Mediators Inflamm..

[13] Jiang Y., Jia T., Wooley P.H., Yang S.Y. (2013). Current research in the pathogenesis of aseptic implant loosening associated with particulate wear debris. Acta. Orthop. Belg..

[14] Abrahamsson I., Berglundh T., Lindhe J. (1998). Soft tissue response to plaque formation at different implant systems. A comparative study in the dog. Clin. Oral Implants Res..

[15] Lang N.P., Bragger U., Walther D., Beamer B., Kornman K.S. (1993). Ligature-induced peri-implant infection in cynomolgus monkeys. I. Clinical and radiographic findings. Clin. Oral Implants Res..

[16] Watzak G., Zechner W., Tangl S., Vasak C., Donath K., Watzek G. (2006). Soft tissue around three different implant types after 1.5 years of functional loading without oral hygiene: A preliminary study in baboons. Clin. Oral Implants Res..

[17] Reinedahl D., Chrcanovic B., Albrektsson T., Tengvall P., Wennerberg A. (2018). Ligature-Induced Experimental Peri-Implantitis-A Systematic Review. J. Clin. Med..

[18] Moest T., Wrede J., Schmitt C.M., Stamp M., Neukam F.W., Schlegel K.A. (2017). The influence of different abutment materials on tissue regeneration after surgical treatment of peri-implantitis—A randomized controlled preclinical study. J. Craniomaxillofac. Surg..

[19] Carcuac O., Abrahamsson I., Albouy J.P., Linder E., Larsson L., Berglundh T. (2013). Experimental periodontitis and peri-implantitis in dogs. Clin. Oral Implants Res..

[20] Rovin S., Costich E.R., Gordon H.A. (1966). The influence of bacteria and irritation in the initiation of periodontal disease in germfree and conventional rats. J. Periodontal Res..

[21] Martins O., Ramos J.C., Baptista I.P., Dard M.M. (2014). The dog as a model for peri-implantitis: A review. J. Investig. Surg..

[22] Baron M., Haas R., Dortbudak O., Watzek G. (2000). Experimentally induced peri-implantitis: A review of different treatment methods described in the literature. Int. J. Oral Maxillofac. Implants.

[23] Nguyen Vo T.N., Hao J., Chou J., Oshima M., Aoki K., Kuroda S., Kaboosaya B., Kasugai S. (2017). Ligature induced peri-implantitis: Tissue destruction and inflammatory progression in a murine model. Clin. Oral Implants Res..

[24] Donath K., Breuner G. (1982). A method for the study of undecalcified bones and teeth with attached soft tissues. The Sage-Schliff (sawing and grinding) technique. J. Oral Pathol..

[25] Johansson C.B., Morberg P. (1995). Importance of ground section thickness for reliable histomorphometrical results. Biomaterials.

[26] Johansson C.B., Morberg P. (1995). Cutting directions of bone with biomaterials in situ does influence the outcome of histomorphometrical quantifications. Biomaterials.

[27] Wilson T.G., Valderrama P., Burbano M., Blansett J., Levine R., Kessler H., Rodrigues D.C. (2015). Foreign bodies associated with peri-implantitis human biopsies. J. Periodontol..

[28] Wang X., Li Y., Feng Y., Cheng H., Li D. (2019). Macrophage polarization in aseptic bone resorption around dental implants induced by Ti particles in a murine model. J. Periodontal Res..

[29] Donath K., Laass M., Günzl H.J. (1992). The histopathology of different foreign-body reactions in oral soft tissue and bone tissue. Virchows. Arch. A.

[30] Mbalaviele G., Novack D.V., Schett G., Teitelbaum S.L. (2017). Inflammatory osteolysis: A conspiracy against bone. J. Clin. Invest..

[31] Paiva K.B.S., Granjeiro J.M. (2017). Matrix Metalloproteinases in Bone Resorption, Remodeling, and Repair. Prog Mol. Biol. Transl. Sci..

[32] Setzen G., Williams E.F. (1997). Tissue response to suture materials implanted subcutaneously in a rabbit model. Plast. Reconstr. Surg..

[33] Nair P.N. (2004). Pathogenesis of apical periodontitis and the causes of endodontic failures. Crit. Rev. Oral Biol. Med..

[34] You T.M., Choi B.H., Zhu S.J., Jung J.H., Lee S.H., Huh J.Y., Lee H.J., Li J. (2007). Treatment of experimental peri-implantitis using autogenous bone grafts and platelet-enriched fibrin glue in dogs. Oral Surg. Oral Med. Oral Pathol. Oral Radiol. Endod..

[35] Schou S., Holmstrup P., Jorgensen T., Stoltze K., Hjorting-Hansen E., Wenzel A. (2003). Autogenous bone graft and ePTFE membrane in the treatment of peri-implantitis. I. Clinical and radiographic observations in cynomolgus monkeys. Clin. Oral Implants Res..

[36] Yu X., Hu Y., Freire M., Yu P., Kawai T., Han X. (2018). Role of toll-like receptor 2 in inflammation and alveolar bone loss in experimental peri-implantitis versus periodontitis. J. Periodontal Res..

[37] Albouy J.P., Abrahamsson I., Berglundh T. (2012). Spontaneous progression of experimental peri-implantitis at implants with different surface characteristics: An experimental study in dogs. J.Clin. Periodontol..

[38] Jovanovic S.A., Kenney E.B., Carranza F.A., Donath K. (1993). The regenerative potential of plaque-induced peri-implant bone defects treated by a submerged membrane technique: An experimental study. Int. J. Oral Maxillofac. Implants.

[39] Hayek R.R., Araujo N.S., Gioso M.A., Ferreira J., Baptista-Sobrinho C.A., Yamada A.M., Ribeiro M.S. (2005). Comparative study between the effects of photodynamic therapy and conventional therapy on microbial reduction in ligature-induced peri-implantitis in dogs. J. Periodontol..

[40] Machtei E.E., Kim D.M., Karimbux N., Zigdon-Giladi H. (2016). The use of endothelial progenitor cells combined with barrier membrane for the reconstruction of peri-implant osseous defects: An animal experimental study. J. Clin. Periodontol..

[41] Abdul-Karim F.W., Benevenia J., Pathria M.N., Makley J.T. (1992). Case report 736: Retained surgical sponge (gossypiboma) with a foreign body reaction and remote and organizing hematoma. Skeletal Radiol..

[42] Puvanesarajah V., Fayad L.M., Rao S.S., McCarthy E.F., Morris C.D. (2019). Extremity gossypiboma mimicking sarcoma: Case report and review. Skeletal Radiol..

[43] Sari A., Basterzi Y., Karabacak T., Tasdelen B., Demirkan F. (2006). The potential of microscopic sterile sponge particles to induce foreign body reaction. Int. Wound J..

[44] Altman G.H., Diaz F., Jakuba C., Calabro T., Horan R.L., Chen J., Lu H., Richmond J., Kaplan D.L. (2003). Silk-based biomaterials. Biomaterials.

[45] Nooh N., Abdullah W.A., Grawish Mel A., Ramalingam S., Javed F., Al-Hezaimi K. (2014). The effects of surgicel and bone wax hemostatic agents on bone healing: An experimental study. Indian J. Orthop..

[46] Spelzini F., Konstantinovic M.L., Guelinckx I., Verbist G., Verbeken E., De Ridder D., Deprest J. (2007). Tensile strength and host response towards silk and type i polypropylene implants used for augmentation of fascial repair in a rat model. Gynecol. Obstet. Invest..

[47] Trindade R., Albrektsson T., Galli S., Prgomet Z., Tengvall P., Wennerberg A. (2018). Osseointegration and foreign body reaction: Titanium implants activate the immune system and suppress bone resorption during the first 4 weeks after implantation. Clin. Implant. Dent. Relat. Res..

[48] Brodbeck W.G., Macewan M., Colton E., Meyerson H., Anderson J.M. (2005). Lymphocytes and the foreign body response: Lymphocyte enhancement of macrophage adhesion and fusion. J. Biomed. Mater. Res. A.

[49] Rodriguez A., Macewan S.R., Meyerson H., Kirk J.T., Anderson J.M. (2009). The foreign body reaction in T-cell-deficient mice. J. Biomed. Mater. Res. A.

[50] Selders G.S., Fetz A.E., Radic M.Z., Bowlin G.L. (2017). An overview of the role of neutrophils in innate immunity, inflammation and host-biomaterial integration. Regen. Biomater..

[51] Jhunjhunwala S., Aresta-DaSilva S., Tang K., Alvarez D., Webber M.J., Tang B.C., Lavin D.M., Veiseh O., Doloff J.C., Bose S. (2015). Neutrophil Responses to Sterile Implant Materials. PLoS ONE.

[52] Laffey J.G., Boylan J.F., Cheng D.C. (2002). The systemic inflammatory response to cardiac surgery: Implications for the anesthesiologist. Anesthesiology.

[53] Chrcanovic B.R., Albrektsson T., Wennerberg A. (2014). Diabetes and oral implant failure: A systematic review. J. Dent. Res..

[54] Chrcanovic B.R., Albrektsson T., Wennerberg A. (2014). Reasons for failures of oral implants. J. Oral Rehabil..

